# “Neither we are satisfied nor they”-users and provider’s perspective: a qualitative study of maternity care in secondary level public health facilities, Uttar Pradesh, India

**DOI:** 10.1186/s12913-015-1077-8

**Published:** 2015-09-27

**Authors:** Sanghita Bhattacharyya, Anns Issac, Preety Rajbangshi, Aradhana Srivastava, Bilal I. Avan

**Affiliations:** Public Health Foundation of India, Plot no. 47, Sector 44, Institutional Area, Gurgaon, Haryana 122002 India; London School of Hygiene and Tropical Medicine, Keppel Street, London, WC1E 7HT UK

**Keywords:** Maternity care, Quality of care, Qualitative study, Health facility, India, Developing country

## Abstract

**Background:**

Quality of care provided during childbirth is a critical determinant of preventing maternal mortality and morbidity. In the studies available, quality has been assessed either from the users’ perspective or the providers’. The current study tries to bring both perspectives together to identify common key focus areas for quality improvement.

This study aims to assess the users’ (recently delivered women) and care providers’ perceptions of care to understand the common challenges affecting provision of quality maternity care in public health facilities in India.

**Methods:**

A qualitative design comprising of in-depth interviews of 24 recently delivered women from secondary care facilities and 16 health care providers in Uttar Pradesh, India. The data were analysed thematically to assess users’ and providers’ perspectives on the common themes.

**Results:**

The common challenges experienced regarding provision of care were inadequate physical infrastructure, irregular supply of water, electricity, shortage of medicines, supplies, and gynaecologist and anaesthetist to manage complications, difficulty in maintaining privacy and lack of skill for post-delivery counselling. However, physical access, cleanliness, interpersonal behaviour, information sharing and out-of-pocket expenditure were concerns for only users. Similarly, providers raised poor management of referral cases, shortage of staff, non-functioning of blood bank, lack of incentives for work as their concerns.

**Discussion:**

The study identified the common themes of care from both the perspectives, which have been foundrelevant in terms of challenges identified in many developing countries including India. The study framework identified new themes like management of emergencies in complicated cases, privacy and cost of care which both the group felt is relevant in the context of providing quality care during childbirth in low resource setting. The key challenges identified by both the groups can be prioritized, when developing quality improvement program in the health facilities. The identified components of care can match the supply with the demand for care and make the services truly responsive to user needs.

**Conclusion:**

The study highlights infrastructure, human resources, supplies and medicine as priority areas of quality improvement in the facility as perceived by both users and providers, nevertheless the interpersonal aspect of care primarily reported by the users must also not be ignored.

**Electronic supplementary material:**

The online version of this article (doi:10.1186/s12913-015-1077-8) contains supplementary material, which is available to authorized users.

## Background

Every year about 287,000 women die due to causes associated with childbirth, and another 10 million suffer from complications related to pregnancy and childbirth [1]. India alone accounts for about a fifth of the global maternal mortality burden [1]. Quality of care provided during childbirth is a critical determinant of increasing utilization of services and preventing maternal mortality and morbidity [2, 3]. Various quality improvement models have been used to assess quality from the users’ perspective [4–8] and also from providers’ perspective [9, 10].

In developing country context, the focus of interventions has been on enhancing service availability. However, maintaining acceptable quality standards is a prerequisite for ensuring effective utilisation of available services [11]. Given this context, understanding user’s satisfaction with services becomes significant as a marker for high quality of care [7]. In addition, provider’s perspective helps in capturing supply side gaps and challenges, which if addressed can lead to quality improvement in the system [5, 12, 13]. Evidence on quality of maternity care based on both user’s and provider’s perspectives would help determine those aspects of care that need strengthening in developing country contexts to support long-term demand and generate significant changes in health-seeking behaviour.

There are various frameworks to assess quality of care from user perspective like categorizing dimensions of care into structure, process and outcome that have established users’ ability to evaluate quality [5, 6, 12]. There are scales to assess patient perception of care capturing dimensions like healthcare delivery, personnel, responsiveness, assurance, communication, discipline and payment of bribes, which have being tested in several countries [5, 6, 8, 13]. The study uses the Hulton et al. framework which was specifically developed for assessing quality of maternity care within institutional contexts from both user and provider perspective [14]. This framework conceptualizes quality from both the user’s experience and providers’ provision with care. [14]. The present study adapted Hulton’s framework and included eight themes applicable to both user and provider so that the common challenges in providing quality care can be identified. This included access and referral, human and physical resource, respect and dignity, privacy, cognitive support, emotional support and cost of care.

There are studies which have separately tried to understand the user’s perspective of care like responsiveness, promptness, inter-personal behaviour and provider’s perspective in terms of availability and competence to deliver care, provision of medicine and supplies, infrastructure. But there are few studies which have tried to combine user and provider perspectives of quality care within the same framework. Thus the present study aims to assess the users and care providers’ perceptions of care to understand the common challenges affecting provision of quality maternity care in public health facilities in India. Users refer to recently delivered women who had utilised the maternity care service at the public health facilities and care providers denote all those who were associated with providing maternity care. By identifying and understanding common challenges, in terms of the difficulties experienced by the users and the obstacles faced by the service providers it may be possible to address these as part of quality improvement programs in respective facilities.

## Methods

A qualitative descriptive study was conducted in a district in the state of Uttar Pradesh in Northern India. The state is positioned among the bottom-line performers in terms of health indicators, with high infant mortality rate (61) [15], maternal mortality ratio (359) [16] and low percentage of institutional deliveries (25 %) [17]. Based on facility data, the percentage of women who faced any delivery related complications like obstructed labour or premature labour in the state was 66 % and around 19 % of women had post-delivery complications like high fever and lower abdominal pain [17]. The study was conducted to understand user’s experiences with obstetric services and the challenges the providers faces in providing maternity care in secondary level facilities. The main focus was on the perception of the respondents on care and hence the study did not dwell deep into the technical aspects of delivery care and the actual process of care. Secondary level health facilities are designated to manage complications with child birth, and have provisions for surgical care, blood transfusion and newborn care. These facilities function as first referral units for the primary level care facilities. Among the three designated facilities in the study district, only two were functioning as secondary level health institutions and were included for the detailed study.

### Study instruments

The data collection was aided by semi-structured, in-depth interview (IDI) guides to understand user’s and provider’s perspectives of maternity care. Open ended questions were supported with probes, wherever necessary. The IDI guides were developed on the basis of a list of factors identified from a review of literature on users’ experience with maternity care in developing countries [18]. Themes of quality of care included were promptness of service such as availability of the health provider and transport to reach the institution; provision of appropriate medical care (primarily medicine); emotional support and privacy provided during delivery; cleanliness and hygiene of the place of delivery; interpersonal behaviour, cognitive support, and faith in the provider’s competence. The instruments were translated into the local language (Hindi) spoken in the study area and were pre-tested in the study area before the instrument was finalised (see Additional file 1).

### Sampling and data collection

In the present study ‘user’ included women who had delivered at the selected facilities and discharged 7–42 days prior to the interview. ‘Care providers’ included medical officer, gynaecologist, anaesthetist and nurses at the facility level (who were directly involved in maternity care) as well as community health workers (who act as a bridge between users and facility). A list of all users who had delivered at selected facilities and discharged within 7–42 days prior to the interview was collected from the respective facilities. From the list, users were selected purposively to include both normal and complicated deliveries. This was done to understand the differences in the perspective of quality of services rendered. The complicated deliveries included the cases where delivery was conducted through vagina using vacuum or forceps and through surgical procedure. This also included the cases where users suffered from immediate postpartum complications like haemorrhage and sepsis. Only users with live births were included in the study. The study team took help of community health workers to track the users. IDIs were conducted till no new significant responses were emerging, so the final sample included 24 recently delivered women.

Similarly, health care providers who were associated with provision of partum and immediate postpartum care were selected from the facility. This included nurses, doctors, gynaecologist and anaesthetist. Those who were available during the field visit and willing to participate in the study were included. Altogether twelve health providers were interviewed for the study. In addition to this, the study also included four female community health workers (Accredited Social Health Activists or ASHAs), who generally accompany users to facilities for delivery and act as a bridge between community and the health system. The study was conducted between April–May 2014.

Since the study aimed at obtaining a deeper understanding of user and provider’s perceptions, in-depth interview was selected as a suitable method to elicit information. Interviews were conducted by the researchers in the local language. All the interviews with users and community health workers were conducted at their residences, whereas the other health care providers were interviewed at their respective facility as per their convenience.

### Data analysis

**Fig. 1:**
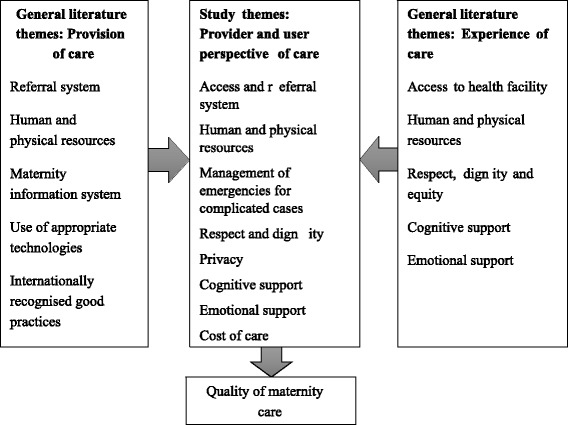
Study framework: Perspective of users and providers on maternity care

Except for a few providers, all respondents allowed audio recording of their interviews. These records were transcribed verbatim and translated into English. Initially ‘a priori’ codes were identified along with emerging themes from the transcripts. Information from users and providers was compared to highlight both their perspectives and the results were arranged according to the eight themes of care derived from the provision and experience of care as stated in the Hulton’s framework (Fig. 1) [14]. The final analysis included eight themes applicable to both user and provider so that the common challenges for both standpoints in providing quality care can be identified. This included access and referral, human and physical resource, respect and dignity, privacy, cognitive support, emotional support and cost of care. Data for each theme was analysed, and codes were provided, categorising user as women who had normal delivery (Wn) and those had complicated deliveries (Wc). The provider data was coded as community health workers (CHW), nurses (Ns), and medical officer, gynaecologist and anaesthetist (Dr).

### Ethical considerations

Ethical approval for this study was granted by the Institutional Ethics Committee of the Public Health Foundation of India (TRC-IEC-187/13). A written permission from district chief medical officer was obtained and the cooperation from head of respective secondary level health facilities was sought. Verbal consent was sought from users whereas a choice between verbal and written consent was given to the care providers. Majority of the providers opted for verbal consent. Anonymity of identity and confidentiality of information was assured to all the participants during analysis.

## Results

### Profile of the respondents

Majority of the users, were illiterate, and belonged to lower caste (Table 1). All the users were unemployed. Majority of the households derived livelihood from cultivation and/or agricultural labour, and the households did not possess any ration card which deprived them from availing benefits of free/subsidised food. Significant proportion of the respondents were educationally, economically, and socially disadvantaged. Majority of the users were multi-para, 13 had normal delivery while 11 had complicated deliveries. Abnormal positioning of foetus, premature labor and prolonged labor contributed to C-section or assisted delivery.

**Table 1:** Study Participants: Profile of health service users

Characteristics		Frequency (*N* = 24)
Age	20 – 25 years	17
	26 – 30 years	6
	Above 30 years	1
Education	No	11
	Yes	13
Religion	Hindu	20
	Muslim	4
Caste	General	3
	Scheduled Caste	12
	Other Backward Caste	9
Occupation of head of household	Cultivator	7
	Agricultural labour	4
	Non-agricultural casual labour	8
	Self-employed outside agriculture	3
	Other	2
Parity	First	11
	Second	6
	Third or more	7
Sex of baby	Male	16
	Female	8
Type of delivery	Normal	13
	complicated	11
Type of complication	Breech	3
	Premature rupture of membrane	3
	Prolonged labour	3
	Sepsis	1
	Post Partum Haemorrhage	1

All providers interviewed were actively involved in the process of delivery and maternity care. The majority were with a total experience in the profession ranging from 5 to 20 years (Table 2). Educational qualification of community health worker- ASHA varied from 8th class to post-graduation.

**Table 2:** Study Participants: Profile of health service providers

Characteristics		Frequency (*N* = 16)
Level of Institution	FRU-District Women Hospital	8
	FRU-Community Health Centre	4
	Community Health Workers	4
Professional Qualification	Gynaecologist	2
	Anaesthetist	2
	Medical Officer	3
	Auxiliary Nurse Midwife (ANM)	5
	Community Health Worker- ASHA	4
Years of total Experience	1–5 years	2
	6–10 years	6
	More than 10 years	8
Sex	Male	3
	Female	13
Caste	General	13
	Other Backward Caste	3

### User and provider’s perspectives

The following section presents the perspectives on the eight study themes.

### Access and referral

The secondary level health facilities are envisaged primarily as first referral units (FRU), equipped to provide specialized care above the primary level health facilities. Hence, apart from providing services to those who live nearby, these facilities cater to those who were referred from lower level facilities. The sub-themes enquired here included availability and mode of transport, choice of facility, and referral process, if any.

### Users’ perspective

Among the users, only five users were referred due to delivery complications from other health facilities. All the other users approached the facility directly due to either proximity, familiarity with the facility or motivation by female community health workers. Another important reason reported for directly approaching the district level facility was the convenience of obtaining the monetary incentive for institutional delivery. Emergency transport services w started in the state few years back. Only nine users could avail these services. In several cases, the availability of ambulance was a problem and users had to wait for more than an hour.

*Pain was increasing…the community health worker called the ambulance service around 11 am. The vehicle came after an hour. I reached the nearby health facility after 30 minutes. I was anaemic and had a breech baby. So they (first facility) referred me to district hospital, which was 50 km away from the first facility. The ambulance took another 1 hour to reach the district hospital*. (Wc.10)

In some other cases, nobody at the helpline received the call. Added to this, a referring health facility did not have an ambulance. So in most of the cases, users had to arrange for own transport which could be either carts, bicycle or motorcycle.

*I was in great pain. My husband called the ambulance at 10 am. But no one picked up the call. Finally, we went by own cart.* (Wc.3)

### Provider’s perspective

With respect to referral, according to the providers there was no coordination between the facilities at lower and higher levels of care. As per the standard care procedure, the users need to be examined and provided immediate management at the primary level facility before sending to another facility. However, the practices on the ground show that these procedures were not followed. Users were either just examined or even tried normal delivery and sent to FRU without any management.

*Women came without any management even at odd hours. Several women came in serious conditions after handled by traditional birth attendants. Further, many women did not have any antenatal care report. Many-a-times they were accompanied by one or maximum two aged females. So making arrangements for medicine, blood and laboratory tests etc. became difficult. Since the CHWs knew the practice, it was rather easy and fast when they accompany the women. (*Dr.1*)*

In the study, CHWs accompanied users to the FRU in 13 cases. The travel time reported by the users varied from 20 min to one hour depending on the distance from the facility. When referred from a community health centre, users generally reached the secondary care facility; but when referred from there to a tertiary care facility, they often defaulted. The users either insisted for conduction of delivery at the FRU or those who could afford opted for private facilities. The reasons quoted include distance, lack of money, lack of transport, and unfamiliarity with the facility and the surroundings. The distance from FRU to tertiary level facility was around 80 km, which may take approximately 1.5 – 2 h of travel by car. The present study was restricted to those users who had delivered at the selected FRUs and did not include those who were referred to a tertiary level facility. The CHWs reported that they were not happy in accompanying users to tertiary level facility.

*I am not familiar with the hospital; I don’t know anyone there. Even if I request, they (staff) do not listen to me. Here (secondary care facility) I know the doctors, nurses, guards, everyone else. I know what to do or where to go. (*CHW1)

From the users’ perspective, along with the distance to reach the facility, non-availability or delay in emergency transport were the main issues in accessing the health facility. Providers perceived the lack of proper management of referral cases by lower level facilities as a challenge for handling complicated cases.

### Physical and human resources

The key concerns covered under physical resources included physical infrastructure of the facility such as waiting area, cleanliness, adequate bedding, staff quarters, constraints in office space and amenities for the providers; regular supply of water and electricity; and medicines and supplies. Workload and availability of facility staff were enquired under human resources. The inadequacies with infrastructure were pointed out by both users and providers.

### User’s perspective

There was no waiting area available for users before admission and for the accompanying persons. Both the antenatal and postnatal care wards were overcrowded. The availability of separate beds was a serious concern to the users. It was not uncommon that two users were sharing a bed. Though the facility had provision for bedding, in several instances, the quality was very poor. Users often carried their own mattresses and bed sheets from home. One female attendant was allowed to stay with the user in the postnatal care ward. However, there was no provision for bedding or seating for the attendant. So they made their own arrangements by sleeping on the floor. There was no provision for accompanying CHWs also. So in case of stay at night, they crowded in front of the emergency room or verandas. They also pointed out the unhygienic conditions of the toilets.

*They (sweeper) clean the ward and toilet once a day. But the toilet is dirty most of the time. Sometimes, women do not pour enough water. Water supply is also not regular.* (Wn.1)

### Provider’s perspective

One of the challenges for providers is irregular supply of water and electricity. In one of the FRU, there was a back-up generator only for the operation theatre and labour room. Even though there was a generator at the other facility, limited funding for fuel constrained its regular use. Therefore, in some cases, deliveries were conducted even in the light of candles. Air conditioning facility was provided in the operation theatre and labour room, but not in the wards. Cleanliness of toilets was reported as a concern by the majority of the users. Due to the irregular supply of water and lack of proper cleaning, some users even restrained from using the toilet. However, the providers blamed the users for unclean toilets and ward.

*Our staffs take regular care in cleaning wards and toilets. However, these village people do not know how to maintain the cleanliness. They make the place dirty.* (Dr.2)

Providers shared concerns of lack of infrastructural facilities like lack of proper office room, rest room, and electricity shortage during peak summers. There is a shortage of accommodation for providers within the facility compound. Some of them were staying nearby while others were commuting 35–40 km every day. The quality and quantity of equipment was another concern.

*There are 10 labour tables in the facility while the average delivery load is 20 per day. Availability of stretcher is limited and many women are shifted to labour room on foot. Trolley is not working properly.* (Dr.1)

Several users reported purchase of medicines, syringes, and cotton pad from outside the facility. Few providers also corroborated the inadequacy in the supply of medicines. Due to increase in the number of women using services, they advised users to make their own arrangements.

*This facility is placed on the border of the block. So the women from the nearby block also utilize our services. We cannot reject them. This causes shortage of medicines*. (Dr.5)

There was a huge shortage of staff reported across the facilities. Normal deliveries were conducted by nursing staff while medical officers and gynaecologist attended complicated cases. Several positions for nurses, gynaecologist and anaesthetist were lying vacant in both the facilities, and the workload was high on the remaining providers.

*16 posts are sanctioned for medical officers, but only five are filled.. no pathologist and paediatrician.. Every staff is overworked. We do not get any leave. Doctors are not willing to join the Government service because of low salary and poor working conditions.* (Dr.2)

*There are 2 nursing staff for a shift. But the work load is too much. Managing women and infants along with documentation work is really hectic.* (Ns.3)

The common challenges reported by both the users and providers include infrastructural constraints, irregular supply of electricity and water, and shortage of medicines and supplies. Heavy workload due to shortage of gynaecologist and anaesthetist, along with inadequate incentives and lack of institutional recognition were specifically pointed out by providers which interfere with delivering quality maternity care. Cleanliness, especially of toilets was a concern for users in fully utilising the services.

### Management of emergency for complicated cases

Round the clock availability of staff, emergency supplies, diagnostic and blood transfusion facilities, and newborn complication management were probed under this theme.

### User’s perspective

In order to avoid the delay in care, the facilities relaxed the registration process and admitted the users soon after arrival. The accompanying CHW or family members completed the registration process after admitting the user. The nurses conducted the examination of users, and only in the complicated cases were the doctors involved. However, the users had mixed experience regarding promptness of care. During the time of the study at one FRU there was no full time gynaecologist, and no functioning facility for blood transfusion. Therefore, they referred all the anaemic and complicated cases (on examination) to the second FRU which was a district hospital. However, there was only one anaesthetist and so they tried to avoid surgery cases during night. Though they had linkage with a nearby blood blank, they generally discouraged blood from there due to some previous mishaps. Unless the user ensured arrangement for fresh blood, they did not admit the case at all. In general, the response was not welcoming for those who came in late evening. The facility staff asked the relatives to take the user to private facility or tertiary level facilities at night. In dire emergencies, either the anaesthetist made a special visit or they referred such cases to tertiary facility.

*My due date was over. I went to the nearby community health centre, but they referred me to the secondary care facility. We reached the health facility at 5 pm. But the labour did not progress even after two hours. The baby was breech. Doctor told my family to consult a medical college, which was 85 km away from the facility. The CHW argued with the lady doctor and insisted that the delivery should be conducted at the facility itself. Finally, the doctor agreed, saying that the facility would not be responsible if anything unpleasant happened. I had an assisted delivery at 10.30 am at the very next day.* (Wc.12)

### Provider’s perspective

The providers mentioned that though it is mandatory for FRUs to have provision for diagnostic and blood transfusion services, none of the facilities had a blood storage unit. The laboratory services and pharmacy were not available round the clock. So those who needed these services during evening or night, had to depend on private services near the hospitals. One facility had a linkage with blood bank in the nearby district hospital. But the facility preferred transfusion only if fresh blood was available. Due to this, most of the anaemic cases who may require blood transfusion were referred to tertiary level facility. Similarly newborn care unit was not fully developed even at the district level facility. So in case of newborn complication, the baby was referred to tertiary facility or the nearby private facility. There was no paediatrician in one of the main facilities; paediatrician from the nearby general hospital attended cases at the FRU during daytime.

*Incubation facilities are limited. For newborn care we do not have radiant warmer, vitamin K cap, laryngoscope, suction cathedrae. We cannot purchase locally because they are not available here.* (Dr.1)

Non-availability of gynaecologist and anaesthetist, especially at night, non-functioning of laboratory and diagnostic services round the clock, and lack of facilities to treat newborn complications were perceived as major constraints for managing complications by both the users and providers. Moreover, the providers emphasized the essentiality of functional blood transfusion facilities, lack of which hinders the very purpose of being a FRU.

### Respect and dignity

Under this broad theme of respect and dignity, the study enquired about the nature of interpersonal behaviour with the facility staff during users’ stay at the facility.

### User’s perspective

Users reported verbal and even sometimes physical abuse by the facility staff. Generally the nursing staff scolded the users when they were shouting in pain. However, users accepted it as normal practice. They even justified this and said that the shouting and moving might cause difficulties for delivery. The nursing staff did not deny this notion. They added that they could not control so many women at the same time in the labour room without applying some authority. The interaction with doctors was limited in majority of the cases. Among the respondents, all the users who had normal delivery were satisfied with the behaviour of doctors. It was mostly those who had complications with delivery who reported that the doctors and other supporting staff behaved arrogantly with them.

*Nobody scolded me. But the way they communicated with us (family) was not good. They get angry easily when somebody requests them to examine the woman.* (Wc.10)

*They told us rudely about the difficulty in conducting normal delivery -if you want the mother and baby, agree to the surgery or take them somewhere else.* (Wc.13)

### Provider’s perspective

Some providers acknowledged that users valued respectful and compassionate behaviour from providers. However, they justified their behaviour in the context of heavy workload and overcrowding.

*We ourselves are exhausted, we are working without any leave, then how can we talk affectionately to women? We too sometimes lose patience.* (Dr.4)

The use of verbal abuse and arrogant behaviour in the facility was widely known to both the users and providers, while only women confirmed physical abuse. Mostly the users with complications explained the details of abusive behaviour since they were in higher need of facility resources and had more interactions with facility staff.

### Privacy

The maintenance of privacy in the specific context denoted three things viz. (i) separate or curtained spaces for examination and delivery, (ii) presence of male providers, and (iii) intrusion/discomfort felt in the presence of males in wards.

### User’s perspective

The users mentioned that there was no dividing screen between labour tables though the labour room was curtained. Some of the antenatal and postnatal wards had curtains while others did not. Except during the visiting time, mostly female relatives were allowed inside the wards. The presence of male relatives of other users was not reported as a privacy concern by the women. However, some users and their family had an objection to male gynaecologists conducting check-up and delivery.

### Provider’s perspective

The challenge the providers faced in providing adequate privacy in form of screens between delivery tables and ward beds was due to infrastructural constraint. Similarly they tried to ensure that unless there was emergency, the users were attended by female doctors.

*People from village do not agree to male doctor examining female patient. The male gynaecologist needs to intervene only during an emergency..* (CHW2)

Users expressed the need for separate and curtained place for examination and delivery. Providers also shared similar concerns; however they reasoned that it was difficult due to overcrowding and infrastructural constraints. The sensitiveness of the gender of the health care provider was acknowledged by both the users and providers.

### Cognitive support

Information sharing, seeking consent, counselling on health of mother and baby and grievance redress mechanisms were the components enquired under cognitive support.

### User’s perspective

Users valued sharing information about their health status. However, explanation of procedures and progression of labour was not communicated to either users or their relatives unless there were some complications.

*They (facility staff) were giving us just instructions… buy this, bring that, do this, do that.... They never explained the reason*. (Wn.3)

Majority of the users could not tell the purpose of any injection, saline and tablets given. Husband’s or relative’s consent was generally sought for conducting C-section procedures only.

*Consent (for C-section) was taken on a form but they (facility staff) did not share the reason for taking the consent… nobody asked whether we wanted to do the operation or not*. (Wc.18)

Users stayed at the facility for 3 days in case of normal delivery and 9 days in case of C-section. There were regular visits by a nurse or doctor in the postnatal care ward. Generally doctors visited once and nurses visited 2–3 times a day. However, very few users received counselling from the facility. Most often, counselling was provided by the nurse on breastfeeding, family planning and vaccination.

### Provider’s perspective

The providers admitted that they were not generally able to provide counselling due to work pressure. They added that they never share any information directly with the user because it might worsen her condition if she feels that the delivery is complicated.

Most of the users as well as providers were not aware of the existence of any mechanism to record users’ feedback about the quality of services. Very few providers spoke about a complaint register or complaint box in the facility. They also acknowledged that women rarely used it.

Users felt the lack of information sharing on health status, progression of labour and other related procedures, along with limited counselling opportunities as factors interfering with the quality of services.

### Emotional support

This theme covered supportive behaviour of facility staff and allowing family member inside labour room.

### User perspective

In the study, the role played by facility staff in calming down users and reassuring support was limited. However, only one user reported that the nursing staff took care in consoling and motivating her during delivery.

*Nurse and dai were very supportive. They told me not to worry and stay calm.* (Wn.11)

In all the other cases this role was performed by CHW or family member. In the case of normal deliveries, CHW and a female relative were allowed inside the labour room. Users perceived this as helpful because the presence of a family member or CHW reduced the chance of verbal and physical abuse, and they provided massage and consolation to the users in pain. A respondent raised the issue of swapping of male babies reported from one of the facility some years before. So they valued the presence of a family member to ensure the safety of mother and baby. For the same reason, a respondent’s family requested entry inside operation theatre, but was denied by the facility staff.

### Provider’s perspective

Though the significance of emotional support was acknowledged by the provider, they assigned it informally to the attendant of the user.

*As soon as the woman enters, we make her to lie down comfortably. We console her that we will be there with her for all support… whatever difficulties arise, we will handle them. We try to keep the woman continue talking even while in pain and we will do our work simultaneously*. (Ns.16)

### Cost of care

The cost incurred for transportation, diagnostic services, medicines and supplies, informal payment, and treatment for newborn and maternal complications were organized under this theme.

### User’s perspective

The care at the public health facility was not all free of cost. The users narrated the expenditure they incurred in availing the services. The registration fees and diagnostic charges were nominal at the facility. Further, the public health facility was supposed to provide all the medicines free of cost to the users. However, many users had to spend a few hundred rupees from their own pocket due to non-functioning of diagnostic services at night, and also due to shortage of medicines and supplies. This expenditure was specifically high for those who had C-section deliveries. For instance, the cost of ultra sound scan at the facility was INR 50 while the same charged INR 400 from a private diagnostic centre. Moreover, there was a wide practice of informal payment. Depending on the sex of the baby, the amount varied from INR 400 to 800. Hence the total cost incurred for a normal delivery was nil to USD 15 while it was up to USD 50 for C-section deliveries.

*Nothing is free there (hospital). If you need care, you have to spend money first.* (Wn.6)

Further, some of the families had to spend several thousand for neonatal service from private hospitals.

*Baby drank bad water (meconium aspiration syndrome). Since no facility was there at the public hospital, baby was taken at night to the nearby private hospital. The care for delivery at public facility and baby’s treatment at private hospital together came around USD 500.* (Wc.7)

The users reported that their expenditure was much more than what the monetary incentive for institutional delivery (under *Janani Suraksha Yojana)*. Previously, the incentive (INR 1400) was distributed as direct cash payment. Due to irregularities this practice was modified and users were paid through cheque. The difficulty with this system was that the majority of the users did not have a bank account and so there was a delay in availing the incentive.

*I will not recommend delivery in that facility. There are high chances that you may lose your life if you do not have money.* (Wc.18)

### Provider’s perspective

Even though some of the providers acknowledged the out of pocket expenditure for medicine and supplies, they did not perceive it as a barrier for users at the public health facility and they felt the cost is much lower than in private facilities.

*All the medicines are provided by the facility. Laboratory tests are carried out free of cost. Ambulance service is free. Food is provided thrice a day. Above all, women are getting monetary incentive for institutional delivery. What to expect more than this?* (Dr.2)

Informal payments, expense for medicines and diagnostic services, and cost for managing newborn complications were pointed out as major cost constraints by the users. Users who had complications spent manifold than their counterparts. However, the providers with the exception of community health workers did not find cost as a barrier to women’s utilisation of services. Instead, the facility based providers highlighted the attractiveness of free services provided at the public health facility along with monetary incentives for institutional delivery as a motivating factor for users. The user’s difficulty in obtaining monetary incentive was shared by community health workers only.

**Fig. 2:**
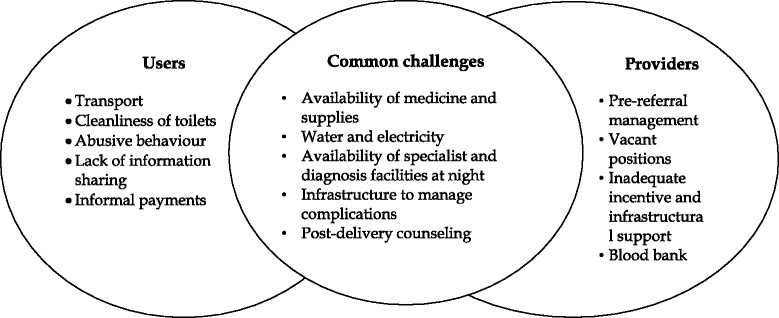
Users and providers’ perspectives of challenges in terms of experience and provision of quality maternity care

From the eight themes arising from in-depth interviews the common challenges highlighted from both the perspectives are presented in Fig. 2.

## Discussion

The study brings out the critical elements of maternity care through the perspectives of both the users and the providers. The providers could explicitly highlight the barriers in providing quality health care, whereas the users expressed their concerns in a more implicit manner. The study highlighted the major themes of care which would only be identified as challenges by users like distance to the facility and difficulty in transportation, cleanliness of toilets, verbal and physical abuses, and arrogant behaviour of providers, non-sharing of information about care, lack of emotional support from facility staff, out-of-pocket expenditure for medicine and supplies, and informal payment for services. The interpersonal aspect of care like respect, dignity and privacy during delivery and better sharing of information could not be comprehended as challenges by providers and they did not consider them as key aspects of care which needed immediate attention at the facility. There are several studies highlighted the significance of interpersonal behaviour and privacy in influencing women’s satisfaction with the facility [8, 19–31].

From the providers’ perspective, issues felt as challenges in provision of quality care refer more to structural aspects such as improper referral of complicated cases by lower level facilities, shortage of gynaecologist and anaesthetist, inadequate incentives and institutional recognition, and lack of blood transfusion facilities. Studies have indicated that adequacy of human resources is essential since a shortage of staff and insufficient skill-mix often resulted in delayed care if not denial of care [8, 23, 32].

Several sub-themes were acknowledged as being common to both the user and provider, such as inadequate physical infrastructure, irregular supply of water and electricity, shortage of medicines and supplies, non-availability of laboratory and diagnostic services at night, difficulties in managing complications during night due to shortage of gynaecologist and anaesthetist, inadequate facilities for managing newborn complications, difficulty in maintaining privacy, inadequate counselling, and complicated procedure for receiving monetary incentive. Similar inadequacies within infrastructure, problems in the supply of medicines and ill-developed emergency management facilities have been reported in studies from other developing countries [33, 34].

Moreover, users were not informed about the procedures and unaware of the technicalities of care at the facility. Providers perceived users as lacking the ability to comprehend, and this led to limited information sharing even during emergencies. Thus the choice offered to the user in terms of procedures was not a free and informed one, but more often a compelled choice. The inadequate information sharing with users has been reported from other developing countries also [31, 35]. The presence of CHWs gains prominence in this context since they have better knowledge about the procedures and facility.

Cost is an important determinant for utilising institutional care. All the users found the cost at public health facility as low in comparison with the cost at a private facility. However, this does not imply that the cost was affordable to all the households. They paid for care which was meant to be free. Considering the poor economic background, many households found difficulty in arranging the money. The expenditure became manifold when the user or baby was referred to another facility due to a shortage of gynaecologist and anaesthetist and lack of technology (especially newborn care and blood bank). Several studies highlighted that the out of pocket expenditure for institutional delivery is higher than the monetary incentive provided by the State [36–38]. Further, the cumbersome process of availing monetary incentive under the new system, if not made user friendly, may reverse the preference for institutional delivery in some cases.

Like certain other countries, [8, 19] physical access to health facility and cleanliness of facility [20] remain a concern in remote areas of the States. The access and referral process gain prominence in the context of timely management of delivery complications. A study conducted in Madhya Pradesh highlighted that the inter facility referrals were poor in quality and lead to adverse birth outcomes [39].

Some challenges were perceived by both the providers and users while some others were predominantly pointed out by one or the other stakeholders. Altogether the challenges mentioned by the stakeholders were complementary and interrelated. Thus an improvement in the quality of care would depend on addressing the concerns raised by both the stakeholders. The priority areas for improvement include developing infra-structure, addressing the human resource shortage, and adequate provision of medicines and supplies. This requires improved budgetary provisioning for healthcare, better human resource management, and inter-sectoral coordination to improve facility based infra-structure as well as rural transportation network. Urgent measures are needed towards equipping the FRUs with sufficient numbers of gynaecologist and anaesthetist, provision of round-the-clock laboratory and diagnostic services, fully functioning blood transfusion facility, and newborn care unit. Interpersonal care in terms of respect, dignity and privacy during delivery and better sharing of information by the providers do not require any major investment, it’s the humane aspect of care that can be easily addressed and provided to the users. This must also not be ignored when delivering quality improvement initiatives.

The study did not find any difference in user’s perception of care on the basis of education and socio-economic status as the study participants had a homogeneous profile. For instance, all the users were either illiterate or having primary level education. Nevertheless, depending on the type of delivery and associated complicates, there was difference in user’s experience at the facility. Users with complicated delivery expressed their concerns over abusive behaviour of staff, high cost incurred for care, difficulties with transport and delay in emergency care.

Even though both the selected facilities were designated as FRUs and were supposed to have provisions for comprehensive emergency obstetric care, yet there were differences with regard to availability of staff (especially gynaecologist and anaesthetist), inadequate capacity for managing emergencies such as lack of generator, availability of diagnostic services, and linkage with blood storage unit. This resulted in multiple referrals and delayed care for users requiring emergency services due to delayed transportation from one FRU to another. A few other studies also pointed out the inadequacies of designated FRUs in India [40, 41].

India’s concern for quality of care in health services has given rise to a series of measures for quality improvement in facilities -ranging from infrastructure norms, accreditation of facilities to community-based monitoring of public health services [42, 43]. Quality, as envisioned in current policies and legislations, is more input-oriented with insufficient focus on outcome and responsiveness to patient’s needs, like courteous behaviour by staff and explanation of diagnosis, treatment and drugs to patients. These aspects of care do not appear to be addressed, and have emerged as one of the major reasons for non-utilisation of public facilities [44]. The adapted framework identified the common themes of care from both the perspectives, which have been found relevant in terms of challenges identified in many developing countries including India [19, 22, 23, 31, 38]. The study framework also identified new themes like management of emergencies in complicated cases, privacy and cost of care which both the group felt is relevant in the context of providing quality care during childbirth in low resource setting [33, 34, 36, 37] . So there is a need to incorporate both the user and provider perspectives in a regular manner to understand quality of services and further research for developing methods to assess maternal satisfaction of care rendered.

### Limitations

The qualitative study design was aimed at obtaining in-depth details of users’ and providers’ perspective. However, the sample comprises users belonging to a younger age group, with low educational levels and lower economic status, all of which can have an implication on their perception. All the users interviewed in the study had live births, so the perception may vary for those who had stillbirth. Another important concern during field enquiry was the ‘courtesy bias’ of respondents. In the study context, this refers to non-reporting of unpleasant facts associated with delivery that was over and also hesitancy to report about their own institutions to non-local investigators. As a result some of the themes of care could not be adequately probed particularly with the providers. The field researchers invested sufficient time in building rapport, clarified the purpose of the interview, assured confidentiality, allowed free talk, and probed wherever necessary. The study also suffers from selection bias as the user and providers were selected in a non- random way. Another limitation of the study was inability to observe the actual delivery care provided, which could have substantiated the challenges that a user and provider narrated in provision of maternity care.

## Conclusion

The study highlighted the common challenges, in terms of the difficulty that the users experienced and the challenges the providers faced in delivering the services. The challenges are apparent in terms of access and referral process, poor management of complicated cases due to inadequate human resource and provision of medicine and supplies. The difference in perspective between users and providers is reflected in connection with respect, dignity and privacy during delivery, sharing of information and cost of care. The key challenges identified by both the groups can be prioritized, when developing quality improvement program in the health facilities. The identified components of care can match the supply with the demand for care and make the services truly responsive to user needs.

## Additional file

Additional file 1:
**In-depth interview guide for study participants.** (DOCX 33 kb)

## Abbreviations

FRU: First referral unit
ASHA: Accredited social health activist
CHW: Community health worker
Ns: Nurses
Dr: Medical officer, gynaecologist and anaesthetist
Wn: Woman who had a normal vaginal delivery
Wc: Woman who had complicated delivery
IDI: In-depth Interviews
