# Supplementary material for: “Neither we are satisfied nor they”-users and provider’s perspective: a qualitative study of maternity care in secondary level public health facilities, Uttar Pradesh, India
Source: BMC Health Serv Res. 2015 Sep 27;15:421. doi: 10.1186/s12913-015-1077-8 (PMC4584124; doi:10.1186/s12913-015-1077-8)
Supplement: Additional file 1: — In-depth interview guide for study participants. (DOCX 33 kb) [file 12913_2015_1077_MOESM1_ESM.docx]

**In- depth Interview Instruments**

**In- depth interview guide for users (recently delivered women)**

1. Demographic details of woman (name, age, religion, caste, education, occupation of respondent/husband/head of household, details of BPL card, place of residence)
2. Kindly provide the following details of your reproductive history (age at menarche, marriage, and first delivery; pregnancy wastage (stillbirth/induced or spontaneous abortion); number of children ever born and number of children surviving (sex-wise); complications with previous delivery, place of previous delivery)
3. Kindly provide details of your current delivery (age & sex of baby, type of institution, nature of delivery, any healthcare services availed during pregnancy (details)? What were your expectations about the baby (health, sex)?)
4. How did you reach the health facility? (Time, distance, mode, availability-especially at night, cost, who assisted & accompanied?)
5. Did you go to any other institution or came directly at the secondary facility? (Explain referral and reason for referral, if applicable) Is this your first visit to the facility? (probe familiarity with facility and providers)
6. Details of woman who had complications during pregnancy/labour/delivery /immediate post-partum period (When and where detected, referral chain, provision for emergency supplies -blood/medicines, availability of skilled staff, how it was managed)
7. What did you do after reaching the facility? (Waiting time, availability of staff, medical examination, who conducted check-ups, diagnostic services, cost, behavior of staff, cleanliness of surrounding, information sharing, privacy)
8. Tell us about what happened when you were taken to labour room? (Who all were present there? Who did what? Probe the time of admission- entry to labour room- delivery, promptness of care - pain management support, medicine and saline, information sharing, family planning services, behavior of staff, cleanliness of labour table, privacy, emotional support by ASHA /family member)
9. How long did you stay at the facility? Kindly narrate what happened during this period. (Monitoring by staff, regular check-ups, complications and responsiveness of staff, medicines and other supplies, food, cost, behavior of staff, cleanliness, availability of water-electricity-clean toilet, counseling/clarifications, privacy, emotional support by ASHA/family, immunization of baby)
10. What happened at the time of discharge? (Was any payment made? Did you have to sign any forms? Any information on further health management provided? If so, who did it & what all were explained? Were you able to understand that?).
11. What was the most important concern to you during delivery? In your opinion, what could be the important needs of a laboring woman?
12. What were your expectations when you came to the facility (in terms of infra-structure, services and provider compared to that in CHC/PHC/SC; information about JSY scheme)?
13. How was your overall experience at the facility? What did you appreciate most at the facility (good infra-structure, presence of MO & ANM, behavior of staff, free tests, no bribe etc.)? What did you find most disappointing? Do you have any suggestions for improvement?
14. Did you avail JSY money (process and problems in availing JSY, if any)?
15. Would you return to the same facility for your next delivery? Would you refer it to others? (Please explain)

********************************************

**In-depth interview guide for providers (Obstetrician-gynecologist/**

**Medical officer)**

**Personal profile**

1. Highest Educational Qualification: ………………………………………………
2. Years of total Experience: ……………………….
3. Experience in the Current Facility: …………….
4. Do you reside in the facility? Does your family also stay with you?
5. Age: ……………… Religion: ………… [a – Hindu; b – Muslim; c – Christian; d – Others]
6. Caste: ……………. [a – General; b – SC; c – ST; d – OBC]

------------------------------------------------------------------------------------------------------------

1. On an average, how many deliveries happen in your facility per month? What are the common complications you come across in your facility? What all can be managed at the facility and what else require referral to tertiary facility (which is the highest facility to which you commonly refer cases)? How many women have complicated/ assisted delivery (need for c- section, use of forceps etc.) in a month? What is the average number of maternal and newborn deaths, and stillbirths in your facility last year?
2. What are the responsibilities of an obstetrician at the facility? (Probe: how many deliveries (and types) you attend in a month?) Is staff conducting delivery available during night also? (Probe: residential facilities for staff; duty hours and emergencies) Tell us whether there is any shortage of staff; if so at what level (nurses; sweepers/ward boys; MOs / specialists / pharmacist/lab technicians?
3. Kindly explain the adequacy of infrastructure of the health facility. [Probe: location of labour room and OT; Issue of sufficient beds in wards, delivery tables, bed sheets / mattresses, equipments, ambulance, toilets, washing facilities, running water, electricity, waiting area for family members; cleanliness in waiting area, ward, labour room, toilets]
4. Whether the availability of medicine/blood is an issue in your facility? If not available in the facility, from where do women/ family members purchase, do you facilitate the process, if so, how? Is drinking water/food available in facility for admitted women? On an average, how much does a woman spend during her stay in the facility (details- food, medicines, diagnostics etc.)?
5. Generally, how long does it take a woman to get admitted for delivery? Are you able to immediately respond to emergency cases? Who monitors the woman in labour room (frequency of your visits to check women in labour)? During check-up and delivery how do you ensure privacy of the woman?
6. How do you comfort a woman in labour? [Probe: Whether the woman is comforted verbally? Presence of a family member during delivery] How do you ensure a comfortable position for delivery? What procedures you usually follow for pain management during delivery? What procedure do you follow for newborns (cleaning, thermal and cord care etc.)? In case of any emergency for newborn how do you handle?
7. How do you mostly communicate information about the woman’s health condition? [Probe: progress of labor; complication; the need for episiotomy, assisted delivery or C-section; adverse outcome] Do you communicate in the local dialect? Do you also talk to the family members about her condition? Are they able to understand the information? Who takes decision in case of any complications (family member/woman/doctor – with/without consent & consultation)? Do women or family members ask any questions or express any demand regarding type of delivery or care?
8. How long is a woman required to stay in the facility (probe by type of delivery); how long does she usually stay? How often is a woman’s condition monitored post-delivery during her stay in the facility? [Probe: no. of visits, information shared]
9. Who generally provides counseling to the mother on postnatal care? If you, then how do you ensure that the woman has understood the given information?
10. What do you think is the most important consideration while providing obstetric care? [Probe: appropriate clinical care, promptness of care, privacy and confidentiality, courtesy, comfort and emotional support, providing adequate information, treating all women the same irrespective of their background] In your experience, what do women value most and feel satisfied with in terms of the care provided?
11. What do you think are the key challenges in providing good quality care in this facility? Is the capacity of the facility sufficient enough to cater to the needs of the population? What are the difficulties that you face in delivering good quality care? Do you have any suggestion to improve the quality of care?
12. In the facility, is there any way to capture women’s satisfaction or dissatisfaction with the services provided? If not, do you think that women’s opinion need to be recorded? If so, how?

******************************************

**In- depth interview guide for providers (auxiliary nurse-midwife /staff nurse)**

**Personal profile**

1. What is your highest completed education/degree? ……… [a – Illiterate; b - Primary school (till 8 standard); c - Secondary school (till 12 standard); d - Graduation and above]
2. How long have you been working as auxiliary nurse-midwife/staff nurse? ……………
3. How long have you been working in the current facility? …………….
4. Nature of appointment: permanent or contractual: ………………………..
5. Do you stay here? Does your family stay with you?
6. Age: ………………… Religion: ………… [a – Hindu; b – Muslim; c – Christian; d – Others]
7. Caste: ……………. [a – General; b – SC; c – ST; d – OBC]

------------------------------------------------------------------------------------------------------------

1. Please tell us about your work. What are your duty hours in a day? Is there a shift system? What are your other responsibilities besides attending deliveries? [probe for time spent on attending deliveries vis-à-vis other responsibilities] Altogether how many nurses are there in the facility?
2. On an average, how many deliveries you attend in a month? How many women have complicated/ assisted delivery (need for c- section, use of forceps etc.) in a month? What are the common complications you come across in your facility? What all can be managed at the facility and what else require referral to tertiary facility? What is the average number of maternal and newborn deaths, and stillbirths in your facility in a year?
3. Who conducts deliveries at the facility (normal / assisted / C-sections)? [Probe: Who handles placenta? Who provides immediate care for the baby? Role of doctor; specialist] Does staff conducting delivery available during night also (Probe: residential facilities for staff)? Tell us whether there is a shortage of staff; if so at what level?
4. Tell us issues regarding infrastructure of the health facility. [Probe: Issue of sufficient beds, delivery tables, cleanliness in the ward and labour room, equipments, ambulance, Toilets, washing facilities, running water, electricity, waiting area for family members]
5. Whether the availability of medicine/blood is an issue in your facility? If not available in the facility, from where do women/ family members purchase, do you facilitate the process, if so, how? Is drinking water/food available in facility for admitted women? Is it free of cost? On an average, how much does a woman spend during her stay in the facility (details – medicine, food, diagnostics etc.)
6. Generally, how long does it take a woman to get admitted for delivery? Who monitor the woman in labor room (frequency)? During delivery how do you ensure privacy of the woman?
7. How do you comfort a woman in labour? [Probe: whether the woman is comforted verbally? presence of family member during delivery] Do you ensure a comfortable position for delivery? What procedures do you usually follow for pain management during delivery? What procedures do you follow for newborn and in case of any emergency for newborn how do you handle?
8. How do you mostly communicate information about the woman’s health condition? What do you communicate to her about? [Probe: who communicates what? progress of labor; complication; the need for episiotomy, assisted delivery or C-section; adverse outcome;] Do you also talk to the family members about her condition? Do you communicate in the local dialect? Who takes the decision in case of complications? Do women ask any questions or express any demand regarding type of delivery or care?
9. How long is a woman required to stay in the facility (probe by type of delivery); how long does she usually stay? How often is a woman’s condition monitored post-delivery within 48 hrs / during her stay in the facility? [Probe: no. of visits, information shared; immunization of newborn]
10. How do you counsel the mother on postnatal care? What are the messages you convey? How do you ensure that the woman has understood the given information? Do you also talk to her family members?
11. While providing care for pregnant women, what is the most important thing that you consider? [Probe: clinical care, privacy, promptness of care, providing comfort and emotional support, provide good information, treat all women same irrespective of her background] In your experience, what do women value most and feel satisfied with in terms of the care provided?
12. While providing care, how do you try to make the whole delivery process a satisfactory experience for the woman? Please cite examples. What are the difficulties that you face in delivering standard care? Do you have any suggestions to improve the quality of care?
13. In the facility, is there any system of capturing women’s feedback (satisfaction or dissatisfaction) with the services provided? If not, do you think that women’s opinion need to be recorded? If so, how?

********************************************

**In- depth interview guide for Accredited Social Health Activist (ASHA)**

**Personal Profile**

1. What is your highest completed education? ……… [a – Illiterate; b - Primary school (till 8 standard); c - Secondary school (till 12 standard); d - Graduation and above]
2. How long have you been working as ASHA? ……………
3. Religion: ………… [a – Hindu; b – Muslim; c – Christian; d – Others]
4. Caste: ……………. [a – General; b – SC; c – ST; d – OBC]

------------------------------------------------------------------------------------------------------------

1. On an average, how many deliveries do you accompany in a month? Do you generally record the health and delivery history of all women in your area?
2. What are the common obstetric complications you come across in your area? What is the average number of maternal and newborn deaths, and stillbirths in a year?
3. What is the preference for institutional deliveries in your area (estimate the proportion of institutional and home deliveries)? If there are home deliveries happening, then what do you think are the key reasons [probe: distance; cost; no one to take care of other kids at home; Dai is available; any cultural factors]? [Probe: How do women get to health facility? Do they have to pay for transport? Is there transport available even at night if there is an emergency?].
4. How many women you referred to FRU CHC/SDH/DH in the last one year? How many of them were complicated cases? [Probe: reasons for referral, difference in quality of maternity care at secondary care facility compared to SC/PHC/CHC, cost of care, women’s compliance]
5. Do women prefer any particular type of facility for institutional delivery? What factors do you think women consider in deciding the place of delivery? [Probe: based on personal experience, the experience of other women, family decision, motivated by the community health worker, awareness of JSY scheme or because of a health complication?]
6. While providing care for pregnant women, what is the most important thing that you consider? [Probe: clinical care, promptness of care, providing comfort, providing emotional support, provide good information especially on birth preparedness, treat all women same irrespective of her background, help during emergency like arranging transport, medicine]
7. In your experience, what do women value most and feel satisfied with in terms of the care provided?
8. Have women ever given you any feedback on their experience of institutional delivery at FRU CHC/SDH/DH? What are the key concerns they’ve expressed?
